# Interactive Evaluation of a Music Preprocessing Scheme for Cochlear Implants Based on Spectral Complexity Reduction

**DOI:** 10.3389/fnins.2019.01206

**Published:** 2019-11-15

**Authors:** Johannes Gauer, Anil Nagathil, Rainer Martin, Jan Peter Thomas, Christiane Völter

**Affiliations:** ^1^Department of Electrical Engineering and Information Technology, Institute of Communication Acoustics, Ruhr-Universität Bochum, Bochum, Germany; ^2^Department of Otorhinolaryngology, Head and Neck Surgery, St. Elisabeth-Hospital, Ruhr-Universität Bochum, Bochum, Germany

**Keywords:** cochlear implants, signal processing, music signal enhancement, spectral complexity, complexity reduction, auditory distortion

## Abstract

Music is difficult to access for the majority of CI users as the reduced dynamic range and poor spectral resolution in cochlear implants (CI), amongst others constraints, severely impair their auditory perception. The reduction of spectral complexity is therefore a promising means to facilitate music enjoyment for CI listeners. We evaluate a spectral complexity reduction method for music signals based on principal component analysis that enforces spectral sparsity, emphasizes the melody contour and attenuates interfering accompanying voices. To cover a wide range of spectral complexity reduction levels a new experimental design for listening experiments was introduced. It allows CI users to select the preferred level of spectral complexity reduction interactively and in real-time. Ten adult CI recipients with post-lingual bilateral profound sensorineural hearing loss and CI experience of at least 6 months were enrolled in the study. In eight consecutive sessions over a period of 4 weeks they were asked to choose their preferred version out of 10 different complexity settings for a total number of 16 recordings of classical western chamber music. As the experiments were performed in consecutive sessions we also studied a potential long term effect. Therefore, we investigated the hypothesis that repeated engagement with music signals of reduced spectral complexity leads to a habituation effect which allows CI users to deal with music signals of increasing complexity. Questionnaires and tests about music listening habits and musical abilities complemented these experiments. The participants significantly preferred signals with high spectral complexity reduction levels over the unprocessed versions. While the results of earlier studies comprising only two preselected complexity levels were generally confirmed, this study revealed a tendency toward a selection of even higher spectral complexity reduction levels. Therefore, spectral complexity reduction for music signals is a useful strategy to enhance music enjoyment for CI users. Although there is evidence for a habituation effect in some subjects, such an effect has not been significant in general.

## 1. Introduction

As a result of technological and surgical advances most cochlear implant (CI) recipients achieve good speech perception in quiet after 6 months of CI use, many of them are even able to make telephone calls (Lenarz, 2018). In cases of profound or total sensory hearing loss CIs allow to restore auditory perception by means of a direct electrical stimulation of the auditory nerve using up to 22 electrodes covering the complete length of the cochlear duct or parts thereof. The number of available electrodes depends on the implant manufacturer and the selected electrode design. The limited number of electrodes and the transmission of electrical currents through the conductive fluid in the cochlear duct cause a spread of excitation where numerous nerve endings associated with a wide range of frequencies are affected by the stimulation from a single electrode (Wilson and Dorman, 2008). The sound coding strategies of CIs are intended to restore speech intelligibility in the first place. They transmit the temporal envelope and the coarse spectral structure of the acoustic signal. The properties of music, however, differ from those of speech in terms of spectral, temporal, and timbral complexity, as well as dynamic range (Limb and Roy, 2014) and can therefore only roughly be represented by state-of-the-art stimulation strategies. As a result of the coarse frequency-to-electrode mapping, the broad excitation patterns, and the limitations of transmitting temporal fine structure information, music perception and music appraisal remain poor for most CI recipients. In particular, CI users face problems with recognition and discrimination of pitch-based and melodic elements of music (Jiam et al., 2017). Additionally, polyphonic melodies which are common in western music are usually perceived as fused (Donnelly et al., 2009) and CI users struggle to distinguish between consonant and dissonant chords (Caldwell et al., 2016). In contrast to pitch-related musical features, the perception of rhythmic features by CI users is comparable to normal-hearing (NH) listeners (McDermott, 2004, Looi et al., 2012, Limb and Roy, 2014). Rhythmic information can be defined as regular temporal patterns with periodicities ranging from 50 ms to 5 s (Nogueira et al., 2019) and thus varies significantly slower than the temporal fluctuations being perceived as pitch. Rhythm is encoded in the slowly varying temporal envelope of pulse trains for the individual electrodes. Therefore, this information does not depend on an exact tonotopy and is thus properly transmitted by the CI.

As a consequence, CI users prefer simple monophonic over complex polyphonic music pieces and more regularly structured genres, such as pop or country music over more complex genres, such as classical music (Gfeller et al., 2003, Looi et al., 2007). In the context of music the term *complexity* is used to describe the lack of structural characteristics or redundancy, such as very simple and repetitive melodic or rhythmic patterns (*objective complexity*). Furthermore, subjective complexity is the result of the interaction between the objective complexity (structural characteristics) of the stimulus and the listener's musical knowledge, prior experience with the musical style and/or idiom, and familiarity with the particular musical stimulus (Gfeller et al., 2003). In contrast to these definitions of *complexity*, in this paper we refer to the definition of the term *spectral complexity* by Hall et al. (2002) and Schönwiesner et al. (2005) as the number of simultaneous spectral components or overtones in a complex tone.

Music listening might be affected by many individual variables, such as the duration of hearing loss, etiology, musical training, listening experience and age (Gfeller et al., 2000). Gfeller et al. (2008) show that pre-implant formal music training is a significant predictor for the appreciation of music with lyrics in CI users. As music plays an important part in everyday life (Lassaletta et al., 2007), improving the appreciation of music in CI listeners is therefore important for successful hearing rehabilitation. Previous works followed different directions to achieve better music perception and appraisal in CI listeners. These encompass music rehabilitation and training as described for instance by Galvin et al. (2009), Looi et al. (2012), and Fuller et al. (2018) as well as novel music compositions (Innes-Brown et al., 2012, Nogueira and Herrera, 2015). Also novel implantation techniques (Hochmair et al., 2015), improved signal coding (Omran et al., 2011; Müller et al., 2012; Todd et al., 2017) and signal preprocessing methods have been proposed.

These latter methods aim at reducing perceptual distortions in CI listeners induced by the shortcomings of electrical stimulation like the spread of excitation and channel interactions between adjacent electrodes. In a pilot study Buyens et al. (2014) investigated the hypothesis that CI listeners prefer a different balance of individual voices and instruments in music signals than NH listeners and presented remixed versions of multi-track pop and rock music recordings to CI users, where vocals, drums, and bass lines were amplified by 6 or 12 dB with respect to the remaining accompaniment. A listening experiment with CI users performing pair-wise comparisons yielded a significant preference for music pieces remixed at 6 dB level difference over the original pieces and the versions remixed at 12 dB level difference, respectively. Similarly, Kohlberg et al. (2015) produced remixes of a multi-track recording of a country music song containing one to five of originally ten instruments and found a significant preference for remixes containing only one to three instruments as compared to the original music pieces in a listening experiment with CI listeners. Moreover, Nemer et al. (2017) showed that reducing the number of overtones in a monophonic melody actually helps CI listeners to perceive music as more pleasant. In contrast to those methods approaches have been proposed that do not rely on multi-track recordings or manual preprocessing: Based on their earlier work the authors of Buyens et al. (2015) proposed source separation and remixing schemes for pop and rock music using harmonic/percussive sound separation (HPSS) to accentuate primarily vocals and drums with respect to other instruments and evaluated them in listening experiments with CI listeners (Buyens et al., 2017). In Pons et al. (2016) and Gajęcki and Nogueira (2018) the authors reported that such an accentuation can also be achieved by means of multi-track source separation using artificial neural networks. They compared different network architectures and also evaluated the obtained remixes with both NH and CI listeners. Cappotto et al. (2018) enhanced the dominant melody of music pieces by adding a continuous-phase sine wave following the fundamental frequency trajectories with variable amplitude.

An alternative approach to reduce the spectral complexity of music signals was introduced by Nagathil et al. (2016) and Nagathil et al. (2017). This method computes segmented reduced-rank approximations in the time-frequency domain based on dimensionality reduction techniques, such as principal component analysis (PCA). While PCA is not the only available technique to achieve spectral complexity reduction, it has the advantage to work fully blind, thus without either prior knowledge of the signals (like the score of a piece), previously learned dictionaries like in non-negative matrix factorization (NMF) or the necessity of prior training as in a neural network. As rhythmic information is known to be well-perceived by CI listeners (Bruns et al., 2016) this method aims at enhancing the spectral representation of music signals and was evaluated for classical chamber music which is not dominated by percussive and other strong rhythmic elements. It was recently also extended for the binaural case, providing an improved attenuation of accompanying instruments (Gauer et al., 2018). The PCA-based spectral complexity reduction method has been evaluated in listening experiments with NH listeners with spectral smearing by broadened auditory filters (Nagathil et al., 2016) and with CI users (Nagathil et al., 2017): the participants rated their preference between an unprocessed and a spectrally reduced version of several music signals in a two-alternative-forced-choice (2AFC) test. In both experiments three different preprocessing methods were compared: PCA, partial least squares analysis (PLS), and the “active-set Newton algorithm” (ASNA, Virtanen et al., 2013). In this context, PLS can be regarded as a score-informed variant of PCA, which puts more emphasis on the melody voice than the fully blind PCA method. The PCA-based method outperformed PLS and ASNA in terms of preference both for NH and CI listeners. Signals both with a moderate (13 retained PCA components) and a higher spectral complexity reduction (8 retained PCA components) have been significantly preferred over unprocessed music. Furthermore, the CI users favored a higher degree of reduction (8 components) slightly more often over the unprocessed signals than those with moderate reduction (13 components). Additionally, the subjects with bimodal hearing showed a higher preference for a moderate complexity reduction while those with unimodal electrical hearing preferred the higher reduction.

The preference for a stronger complexity reduction found in unimodal CI listeners and the fact that high reduction levels had not been studied before led to the main research question of this study, i.e., to investigate the impact of a wider range of complexity reduction levels on the preference of CI users. Hence for the listening experiments presented here we extended the number as well as the range of spectral complexity reduction levels from three (unprocessed, 8 and 13 retained PCA components) to ten (unprocessed, 20, 15, 10, 8, 7, 6, 5, 4, 3 components). For this number of conditions paired comparisons are no longer feasible. Therefore, instead of a 2AFC test we developed a new interactive experimental setup that enables the participants to compare different complexity settings intuitively and in real-time. Moreover, to examine interindividual factors which might have an influence on the preferred spectral complexity reduction level we included different questionnaires dealing with musical engagement as well as musical abilities and the history of hearing loss. As a secondary research question we investigated possible long term effects by performing the listening experiments with partly varying stimuli in eight consecutive sessions. We formulate the research hypothesis that repeated engagement with music signals of reduced spectral complexity leads to a habituation effect such that CI users gain improved enjoyment also for music signals with a higher level of spectral complexity.

## 2. Methods and Materials

### 2.1. Signal Processing for Spectral Complexity Reduction

#### 2.1.1. Spectral Complexity Reduction Method

The signal preprocessing method proposed by Nagathil et al. (2016) is based on the assumption that in signals with competing voices the spectrum will show the strong partial tones of a predominant melody or leading voice as its most prominent elements. These spectral elements can be identified block-wise using principal component analysis (PCA) on short-time spectral representations of music signals. By reconstructing a music signal only from a limited number of its first principal components we achieve a reduction of the spectral complexity which is expressed in a reduction of weaker overtones in general and usually results in an attenuation of the accompanying voices. From an algebraic point of view this approach corresponds to a dimensionality reduction and a block-wise reduced-rank approximation of the original signal.

The computation of the PCA-based reduced-rank approximation can lead to a low-pass filter effect such that the processed signals sound somewhat muffled, especially when only a low number of components (5–10) are retained. Therefore, the mixed music signals were initially fed to a first-order pre-emphasis filter that alleviates this low-pass filter effect and compensates for a spectral tilt toward higher frequencies. After processing the original spectral tilt was restored by a corresponding de-emphasis filter. Then, a spectral representation was computed by means of a sliding-window constant-Q transform (CQT, Brown, 1991) in the frequency range between 55 and 7,040 Hz using a frame shift of 2 ms, two frequency bins per semitone, and Hann analysis windows. In total, this resulted in 168 CQT frequency bands corresponding to seven octaves. The CQT time-frequency representation of the full signal was segmented into *M* half-overlapping blocks **U**^(*m*)^∈ℂ^*K*×*B*^, with *m* = {0, 1, …, *M*−1} being the block index, *B* denoting the number of time frames in one block, and *K* being the number of frequency bands. For notational convenience the block index *m* is dropped in the following. In accordance to the parameter settings in the previous work by Nagathil et al. (2016) a block length of *B* = 100 frames, corresponding to 200 ms, was chosen and the number of frequency bands was *K* = 168. Then, the first *L* < *K* eigenvectors of the covariance matrix **C** ~ **UU**^H^ were computed, which were stored as column vectors of the matrix **W** = ℂ^*K*×*L*^. The resulting principal component scores were computed as the mapping **S** = **W**^H^**U**. Exploiting the orthogonality of the eigenvectors, a rank-*L* approximation of **U** was obtained by $\hat{\text{U}}=\text{W}\text{S}=\text{W}{\text{W}}^{\text{H}}\text{U}$. This procedure was repeated for each time–frequency block **U**. The simplified time–frequency representation of the whole signal was obtained by overlap-adding consecutive processed blocks $\hat{\text{U}}$. For the reconstruction of a signal from the (modified) CQT spectrum the method by Nagathil and Martin (2012) was applied, which relies on short, half-overlapping synthesis frames of 4 ms length and recovers the full-length signal using the overlap-add method with a Hann synthesis window. To reverse the effects of the pre-emphasis filter, the reconstructed signal was fed to a corresponding first order de-emphasis filter.

**Figure 1:**
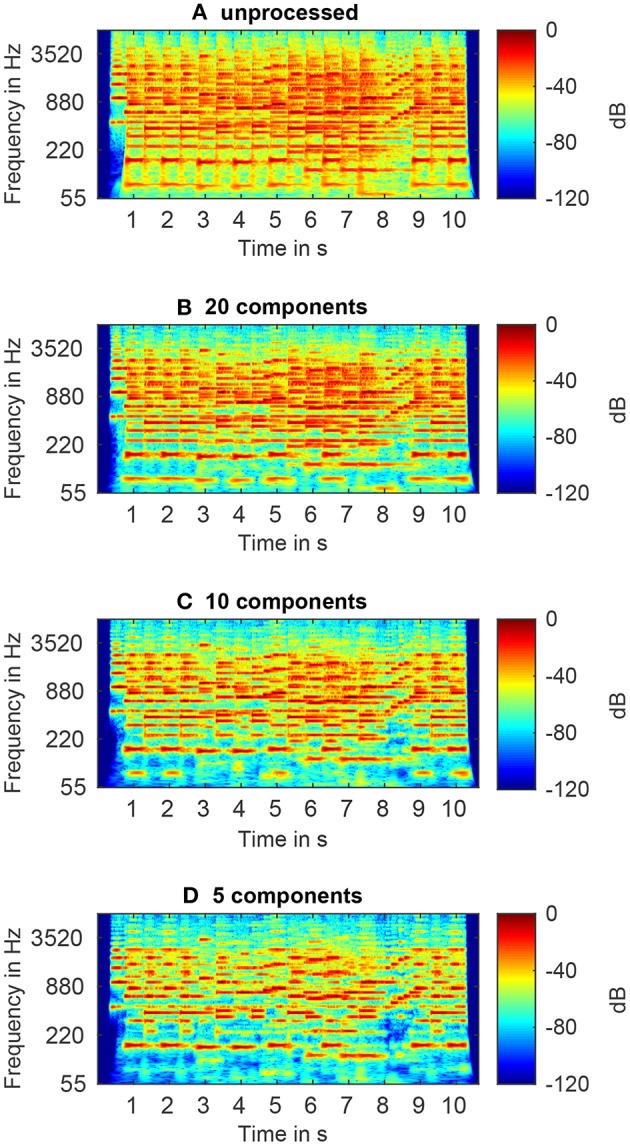
CQT spectrograms of an unprocessed signal **(A)**, and spectrally reduced signals with 20 **(B)**, 10 **(C)**, and 5 **(D)** retained components.

CQT spectrograms of a chamber music piece before and after PCA-based spectral complexity reduction with 20, 10, or 5 retained components are shown in Figure 1. It can be seen that these reduced-rank approximations attenuate low-energy harmonics of both leading voices and accompaniment and, thus, achieve an effective reduction of the spectral complexity.

#### 2.1.2. Instrumental Assessment of Spectral Spread Improvement

The Auditory Distortion Ratio (ADR) measure proposed by Nagathil et al. (2016) was developed to instrumentally evaluate changes due to reduced frequency selectivity and spectral spread between processed and unprocessed signals (see also Nogueira et al., 2019). It is based on broadened auditory filters that mimick the reduced frequency selectivity in listeners with severe hearing loss. These broadening filters can be described using the auditory model introduced by Moore et al. (1992). Regarding the electric stimulation in cochlear implants we observe similar effects of a reduced frequency selectivity and spectral spread. This spectral spread is caused by the spread of excitation that occurs due to the non-focussed electrical field within the cochlear duct between the particular stimulating electrode and the adjacent nerve fibers. Comparable to the distortion induced by overdriven or clipping electrical devices, broadened auditory filters also introduce higher-order harmonics. The distortion of these harmonics which is measured by the ADR is relatively weak, therefore the resulting ADR values in dB are rather small.

### 2.2. Participants

The inclusion criteria for this study comprised unilaterally or bilaterally implanted CI adult users (age ≥ 18 years) with a post-lingual hearing loss, CI experience of at least 6 months and without severe cognitive or neurological impairments. Ten adults (seven female, three male) aged from 47 to 79 years (median 69 years) participated in the listening experiments. Table 1 gives an overview of participants' gender and age as well as their etiology of hearing loss and post-operative speech perception, their duration of CI experience and their provided devices and coding strategies. Two participants were bilaterally implanted. Among the remaining subjects four were implanted only on the left and four only on the right ear. The data in Table 1 refers to the side used during the listening experiments. Most of the subjects suffered from a progressive hearing loss over the last 10–30 years, one participant (P09) since adolescence. Those subjects that have not been implanted bilaterally were suffering from a moderate to severe hearing loss also on the contralateral ear and wore an additional hearing aid, except subject P10 who has been provided with a bone conduction implant on the contralateral side. Speech perception in quiet was assessed using the Freiburg monosyllabic word test (Hahlbrock, 1953, Müller-Deile, 2009) at 65 dB and at 80 dB with a mean speech intelligibility of 61.0% at 65 dB and of 78.5% at 80 dB, respectively.

**Table 1:** Demographic, etiological, and CI-specific information about the 10 CI listeners participating in the listening experiments.

**Subject**	**Gender**	**Age**	**Etiology**	**Speech perception at 65/80 dB (%)**	**CI experience (years)**	**Sound processor and implant**	**Coding strategy**
P01	f	51	Cholesteatoma	50/95	5	Sonnet, Sonata Standard (l)	FSP, FS4
P02	m	64	Chronic otitis media	70/95	4.3	Opus 2, Sonata Standard (l)	FS4
P03	f	73	Sudden hearing loss	65/80	1.2	Sonnet, Sonata Standard (r)	FS4
P04	f	70	Sudden hearing loss, Ménière's disease	75/95	1.4	Naida Q9, HR 90K Mid Scale (r)	HiRes Optima-S
P05	f	62	Sudden hearing loss	65/75	1.9	Naida Q7, HR 90 K Mid Scala (r)	HiRes Optima-S
P06	m	68	Progressive	60/80	2.1	Sonnet, Sonata Flex 28 (r)	FSP
P07	f	71	Sudden hearing loss	50/60	1.5	Opus 2, Combi 40+ (r)	CIS
P08	m	74	Basal skull fracture	55/70	0.5	Sonnet, Synchrony Standard (l)	FS4
P09	f	79	Cholesteatoma	40/50	2.1	Sonnet, Sonata Standard (l)	FSP
P10	f	47	Surditas, Ménière's disease	80/85	2.0	Rondo, Sonata Flex 28 (l)	FSP

The individual center frequency assignments of the CI filterbanks for all participants are depicted in Table 2. All but one of the subjects provided with a Med-EL device (P07) used a fine structure coding strategy (FSP, FS4). For the majority of the subjects the center frequencies of the electrode channels covered the frequency range from 120 to 7.5 kHz, whereas the remaining participants used either the CIS strategy (P07) or the HiRes Optima strategy (P04, P05) with a minimum center frequency of 333 Hz for the first electrode channel.

**Table 2:** Minimum and maximum filterbank frequencies (*f*_min_ and *f*_max_) and estimated tonotopic frequencies at the position of the first three electrodes.

**Subject**	**f_min_/Hz**	**f_max_/Hz**	**f_C1_/Hz**	**f_C2_/Hz**	**f_C3_/Hz**
P01	125	7,326	179	305	481
P02	120	7,410	–	–	–
P03	149	7,412	179	305	481
P04	333	6,665	–	–	–
P05	333	6,665	–	–	–
P06	154	7,328	402	563	823
P07	357	4,741	–	–	–
P08	154	7,328	–	–	–
P09	120	7,410	174	299	473
P10	149	7,412	420	604	849

*The frequencies f_C1_, f_C2_, and f_C3_ refer to the first, second and third electrodes, respectively. The electrode positions are derived from the cochlear duct length measured from CT scan images. For subjects P02, P04, P05, P07, and 08 the required CT scans were not available or only in a quality insufficient for cochlear duct length measurements*.

### 2.3. Selection of Music Stimuli

The complexity reduction method evaluated in this study aims at a spectral complexity reduction and thus mostly effects pitch- and overtone-related features of music signals. To focus on these features, we restricted the choice of musical genres to classical chamber music pieces, a musical genre where harmonic properties are most important. Rhythmic elements, especially represented by percussion instruments, only play a less prominent role here, unlike in genres like pop, rock, or jazz music. In accordance with previous works by Nagathil et al. (2016) and Nagathil et al. (2017) a total number of 16 music stimuli were used in this study. The set contained excerpts of classical chamber music pieces of 10 s with a well-defined monophonic leading voice (clarinet, flute, oboe, trumpet, or violin) and an accompaniment (bassoon, piano, or strings).

A wide range of musical properties were found to have an impact on music perception in CI listeners in previous studies. To allow for an automated analysis of such musical properties, the music pieces were available as MIDI files. We developed a Plackett-Burman experimental design (Plackett and Burman, 1946) that accounts for these properties and selected the stimuli from an original database of 110 pieces based on the following three criteria for the leading voice: the fundamental frequency of the leading voice (Gfeller et al., 2002), the interval size between successive leading voice tones (Sucher and McDermott, 2007), and the tone duration which is connected to the tempo (Vannson et al., 2015). The Plackett-Burman experimental design is used to investigate the dependence of a random variable on a number of independent factors using a minimum number of experiments. For selecting the excerpts from the original music database, temporal averages of these factors across the stimulus duration were calculated, i.e., the mean interval size (MIS), the mean fundamental frequency (MFF), and the mean tone duration (MTD), which were assigned to a “low” and a “high” level, respectively. These levels correspond to the following value ranges: MIS_low_ ≤ 3 semitones and MIS_high_ ≥ 4 semitones, 147 Hz ≤ MFF_low_ ≤ 588 Hz and 698 Hz ≤ MFF_high_ ≤ 2, 792 Hz, MTD_low_ ≤ 0.3 s, and MIS_high_ ≥ 0.5 s.

After the selection process, the MIDI files were synthesized in *Steinberg Cubase* (Steinberg Media Technologies GmbH, Hamburg, Germany) using *Native Instruments Komplete 9* (Native Instruments GmbH, Berlin, Germany) samples which contain note-wise recordings of real instruments. Hence, the synthesized MIDI files had a sound quality similar to that of real-world recordings. The resulting leading voice and accompaniment signals were converted to mono, resampled at 16 kHz, and mixed at equal power.

### 2.4. Experimental Setup

The design of our study combined repeated listening experiments with at-home listening tasks and corresponding questionnaires over a period of 4 weeks. While the listening experiments served to further investigate the preferred spectral complexity reduction in CI listeners as the main research question, we additionally used four different questionnaires to assess the general self-perceived listening quality, the music listening habits and the musical abilities of the participants.

#### 2.4.1. Assessment of Music Listening Habits and Musical Abilities

Music perception is more subjective than speech understanding or localization tasks and also relies on previous knowledge and previous experience by the listeners. Also there is a large variation in musical skills, listening expertise and the abilities to play a musical instrument or to communicate about music (Müllensiefen et al., 2014). Therefore, before the first experimental session the general self-perceived sound quality, the music listening habits and the musical abilities of the participants were assessed using the following questionnaires and methods. The results of these questionnaires delivered additional data to examine possible preconditions for the individual spectral complexity preferences. Thus, the questionnaire results were compared both with the results from the listening experiments and among each other. Correlation analysis was applied to investigate possible predictors of the preferred spectral complexity reduction resulting from the listening experiments.

##### 2.4.1.1. HISQUI

The Hearing Implant Sound Quality Index (HISQUI29) developed by Amann and Anderson (2014) assesses the self-perceived level of auditory benefit in everyday listening situations by means of a 29-item questionnaire scored on a 7-point Likert scale. The total score of maximum 203 is divided into 5 groups (0–60: “very poor sound quality”; 60–90: “poor sound quality”; 90–120: “moderate sound quality”; 120–150: “good sound quality”; 150–203: “very good sound quality”).

##### 2.4.1.2. Gold-MSI

To assess self-reported musical skills and behaviors, a German version of the Goldsmiths Musical Sophistication Index (Gold-MSI) measuring the musical sophistication by 38 different items was used (Müllensiefen et al., 2014; Schaal et al., 2014). Its subscales cover emotional engagement with music, the self-reported singing abilities, the amount of musical training received, the self-reported perceptual abilities, the active musical engagement and the general musicality. Based on the subscale results a General Musical Sophistication score ranging between 18 and 126 can be calculated.

##### 2.4.1.3. Mini-PROMS

The Mini-PROMS test is a computer-based online test which comprises 36 items with subtests on melody, tuning, tempo and accent that are based on pair-wise comparisons of acoustical stimuli. It is a short version of the PROMS test battery developed by Law and Zentner (2012) and was used to assess the musical perception skills of the participants (Zentner and Strauss, 2017). Summarizing the individual subtests, the Mini-PROMS test also provides a total score with a range between 0 and 36.

##### 2.4.1.4. Munich Music Questionnaire

The Munich Music Questionnaire investigates the music listening habits of CI recipients and comprises 25 questions covering music activities both before and after cochlear implantation (Brockmeier et al., 2007). Out of the 25 main questions, four items relevant for this study have been selected: “How often did you listen to music before your hearing loss/with your hearing loss prior to receiving your cochlear implant (CI)/now, after receiving your CI?”, “How does music generally sound with your cochlear implant?”, “How would you rate your enjoyment when listening to music now?” and “Have you practiced listening to music with your implant?” The first three items are assessed by 10-point Likert scales. For the item “How does music generally sound with your cochlear implant?” the sound impression is assessed using the following scales: natural vs. unnatural, pleasant vs. unpleasant, distinct vs. indistinct, less tinny vs. more tinny and less reverberant vs. more reverberant. The item “How would you rate your enjoyment when listening to music now?” comprises the scale “great enjoyment” vs. “no enjoyment” for each of the genres: classical music, opera/operetta, religious music, folk/country music, pop, rock, jazz/blues and “music to dance to.”

#### 2.4.2. Setup of Listening Experiments

Each subject participated in eight consecutive sessions of listening experiments within about 4 weeks. During these sessions the participants listened to a selection of music excerpts of 10 s duration each repeated in an infinite loop. They could modify the level of spectral complexity reduction themselves in real-time using a jog-dial and were asked to choose their preferred spectral complexity reduction level out of ten different levels: original (unprocessed), 20, 15, 10, 8, 7, 6, 5, 4, and 3 retained PCA components. These 10 levels were labeled from 1 to 10 with complexity reduction level 1 corresponding to the unprocessed signal and complexity reduction level 10 corresponding to maximum spectral complexity reduction (3 components). The participants were neither informed about the meaning of the level labels and the differences between the individual signal versions they should choose from, nor about the concept of spectral complexity reduction in general. This assignment of complexity reduction levels was chosen because for small values of retained components there is a considerable difference between the reduced signals to adjacent numbers of retained PCA components, which is also clearly audible (at least for normal-hearing listeners). For higher numbers of retained PCA components the spectrally reduced signals quickly converge toward the unprocessed signal so the differences become increasingly difficult to notice even for normal-hearing listeners. In contrast to typical sound quality assessment tests like, e.g., MUSHRA, the order of the spectral complexity reduction levels in the user interface was not randomized because arbitrary changes in spectral complexity would have led to an unnatural hearing impression while switching between the different complexity reduction levels with the jog-dial during the continuously looped playback. In the take-home evaluation of a preprocessing method conducted by Buyens et al. (2017) the participants also chose their preferred degree of attenuation from a continuous scale.

**Figure 2:**
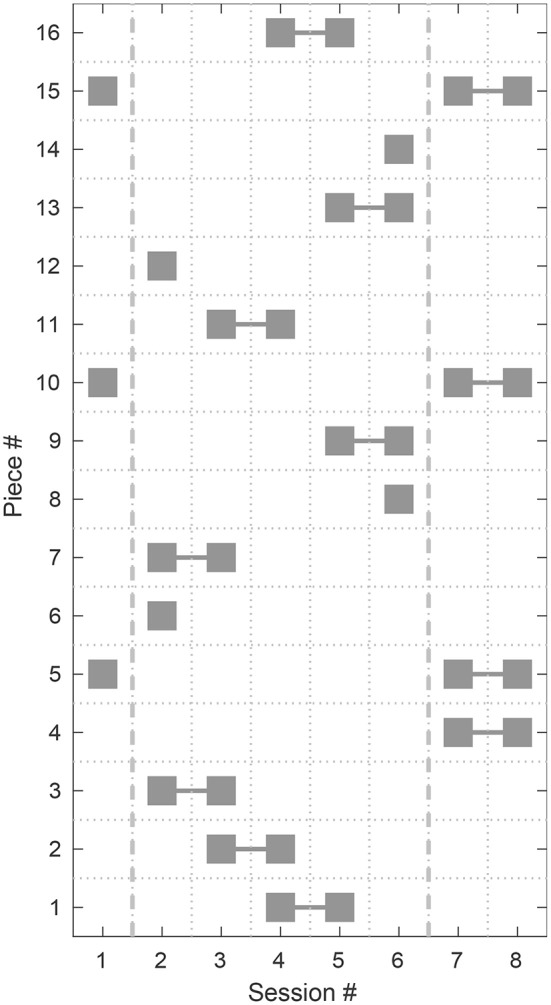
Distribution of the musical stimuli to the particular experimental sessions. The pieces presented in Session 1 were repeated in Sessions 7 and 8 to investigate long term effects. In the Sessions 2 to 6 (between the dashed vertical lines) two pieces from the previous session were repeated and two new pieces were presented in each session.

In the initial session (session 1), three pieces were presented, which were repeated again in the final sessions (session 7 and 8). The repeated tests on these pieces aim to investigate long term effects on the preferred number of retained PCA components over the complete time span of the study. In each of the intermediate sessions 2–6 four pieces out of 16 were presented. In each session two previously presented pieces were presented a second time and two were replaced by new ones (see the distribution scheme in Figure 2). The pieces were assigned to the sessions such that in every session each level (*low* and *high*) of the musical factors MIS, MFF and MTD from the Plackett-Burman experimental design appears twice among the four pieces.

**Figure 3:**
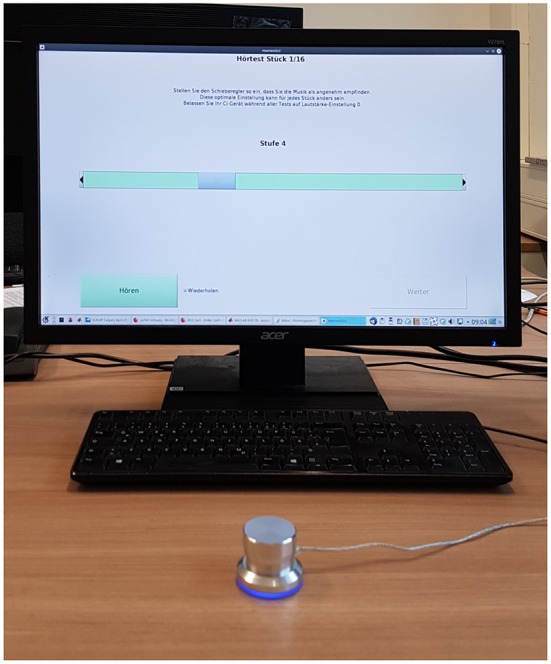
Interactive user interface used in the listening experiments. The participants used the jog-dial in front to select the complexity reduction levels in real-time while the stimuli were presented in an infinite loop. The blue slider on the green bar indicates the currently active level.

During the listening experiments the stimuli were selected from a database of pre-processed signals with different spectral complexity levels using a custom-made graphical user-interface written in MATLAB (The MathWorks, Inc., Natick, MA, USA) and a *Griffin Technologies PowerMate* jog-dial (Griffin Technology, Irvine, CA, USA). The participants used the jog-dial to seamlessly switch between adjacent spectral complexity settings in real-time while the currently selected complexity reduction level (but not the number of retained PCA components) was shown on the display (see Figure 3).

To ensure that the preference ratings were only based on the CI implanted ear, the playback devices were connected directly to the speech processor bypassing their microphones during the experimental sessions, the Mini-PROMS test and the at-home listening tasks. Thereby the influence of background noise, acoustical properties of different playback devices and inappropriate acoustic environments especially during the at-home listening tasks were eliminated. For the MED-EL recipients the *MED-EL direct audio input cable for external sources*^1^ (MED-EL Elektromedizinische Geräte Gesellschaft m.b.H., Innsbruck, Austria) was used to present the stimuli. For the Advanced Bionics recipients the stimuli were presented via the induction loop using the *Phonak ComPilot Accessory*^2^ (Phonak AG, Stäfa, Switzerland) as a direct input is not available here. In both cases the devices were connected to a *Lake People Phone-Amp G109* (LAKE PEOPLE electronics GmbH, Konstanz, Germany) headphone amplifier with fixed signal output level during the listening experiments. In case of the bilaterally implanted listeners (P02, P07) the stimuli were applied to the side implanted earlier and thus with the longer CI experience.

#### 2.4.3. At-Home Listening Tasks

In order to motivate the participants to listen to the music pieces at least 20 min per day between two sessions, home work listening tasks were given: they were instructed to listen to full length recordings of the four pieces presented in the previous session that were processed with the individually preferred level of spectral complexity from the previous experimental session. Additionally, the participants were asked to answer questions in another questionnaire especially designed for the study by a professional musician. It comprised questions on sound perception, character, tempo and the kind of instruments used in the particular piece. This questionnaire was included to make sure that the participants performed the at-home listening tasks. It did not serve to answer any actual research questions. Thus, its results are not relevant for the research questions in this work and will therefore not be presented.

### 2.5. Statistical Evaluation

Both the numbers of retained PCA components selected for evaluation in the listening experiments and the assigned spectral complexity reduction levels form ordinal scales but a proportional relation between the particular elements on these scales cannot be assumed. Therefore non-parametric tests are used to analyze the statistical effects of different factors, such as the participating subjects and the pieces from the database. These tests also do not require the assumption of normal distributions on the data.

Where due to particular factors independent samples can be supposed, the Mann-Whitney U test (also called Wilcoxon rank sum test) was applied. For instance, this is the case with the three factors from the Plackett-Burman experimental design (MIS, MFF, MTD, see section 2.3) that each divide the 16 pieces used in the listening experiments into two non-overlapping subsets. The significance of differences for repeated measurements (pieces presented for the first vs. the second time) was evaluated using the Wilcoxon signed-rank test. The statistical significance of linear regressions of the experimental results was evaluated using the analysis of variance (ANOVA) method. Correlation analysis was performed using non-parametrical measures for ordinal data like Spearman's ρ and Kendall's τ. All statistical tests were performed using built-in functions of MATLAB R2018b.

## 3. Results

### 3.1. Questionnaires

**Figure 4:**
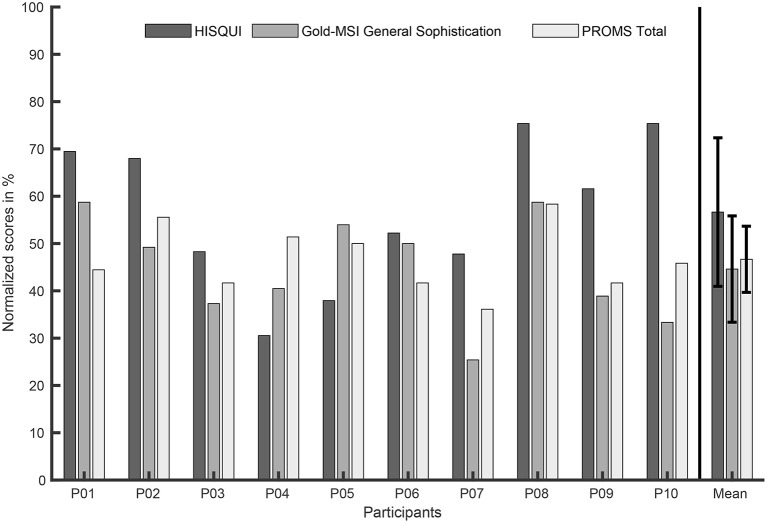
HISQUI, Gold-MSI General Sophistication, and PROMS Total scores per participant as main results from these questionnaires. The very right bars depict the mean and the standard deviation of each score for all participants. All scores were normalized to the maximum values of their respective scales (HISQUI: 203, Gold-MSI: 126, PROMS: 36).

Figure 4 shows the main scores of the HISQUI, Gold-MSI and Mini-PROMS tests. All scores in the figure have been normalized to the maximum values of their respective scales.

#### 3.1.1. HISQUI

The total score of all participants was in a range between 62 and 153 with a scale maximum of 203. Corresponding to the obtained scores two subjects (P08 and P10) reported a “very good,” three (P01, P02, and P09) a “good,” three (P03, P06, and P07) a “moderate” and only two (P04 and P05) a “poor hearing quality.” The mean score of 115 corresponds to a moderate hearing quality.

#### 3.1.2. Gold-MSI

In this study the participants obtained significantly lower scores for all of the subscales (Active Engagement, Perceptual Abilities, Musical Training, Singing Abilities and Emotions) compared to the German reference data for normal-hearing listeners published in Schaal et al. (2014). The General Musical Sophistication score of the participants ranged from 32 to 74 on a scale with possible values between 18 and 126. The participants obtained a mean score of 56.5 compared to 70.4 in the reference data. While the deviation in the mean Singing Abilities score was only moderate (24.1 vs. 27.6), the participants reached a noticeably lower Musical Training score (12.1 vs. 22.6). The Active Engagement and Perceptual Abilities scores were in a range of 13–37 and of 17–43 (with mean values of 24.3 and 33.6 compared to 33.0 and 45.8 reported in the reference data, respectively).

#### 3.1.3. Mini-PROMS

Compared to the data published by Zentner and Strauss (2017) which refers to 152 normal-hearing subjects between 16 and 63 years old and partly amateur or professional musicians, the musical abilities of the participants included in our study are quite low. The participants obtained a mean Total Score of 16.8 vs. 24.56 on a scale of maximum 36, which corresponds to 68.4% of the reference data. Only two of the ten subjects obtained a total score of 20 or more, whereas four participants obtained a total score of 15 or even less. In comparison to the published data the poorest results have been obtained for the “Melody” subtest (47.4%), and the best ones for the “Tempo” subtest (81.3%), whereas the “Tuning” and “Accent” subtests were in between. These results are in line with earlier studies e.g., by Gfeller et al. (2002) and Looi et al. (2004) which showed that pitch and timbre perception is impaired in CI users whereas rhythmic cues can be detected similarly well as by NH listeners.

#### 3.1.4. Munich Music Questionnaire

**Figure 5:**
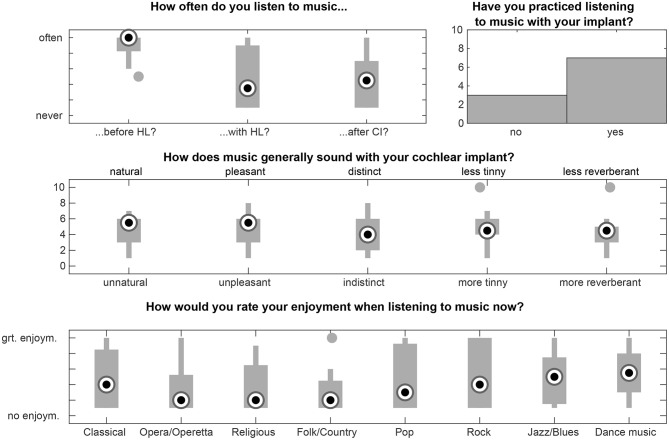
Results from the Munich Music Questionnaire for the selected items “How often did you listen to music before your hearing loss/with your hearing loss prior to receiving your cochlear implant (CI)/now, after receiving your CI?”, “How does music generally sound with your cochlear implant?”, “How would you rate your enjoyment when listening to music now?” and “Have you practiced listening to music with your implant?” The participants filled out a German version of the questionnaire. The items and labels used here are taken from the English questionnaire. Filled circles denote outliers.

Figure 5 depicts the results from the Munich Music Questionnaire. They range on a scale between 1 and 10 (except the question about music listening practice which only allows yes/no answers). According to the questionnaire the participants listened to music quite often before the hearing loss (mean 8.6). After hearing loss the mean score dropped down to 5.0. Cochlear implantation did not significantly change the situation (5.4).

The general impression of music was rated on the scales natural vs. unnatural (mean 4.6), pleasant vs. unpleasant (mean 4.7), distinct vs. indistinct (mean 4.2), less tinny vs. more tinny (mean 4.7) and less reverberant vs. more reverberant (mean 4.4). While some subjects reported to enjoy listening to classic music, others stated to not appreciate it at all. In general, pop, rock, jazz/blues and “music to dance to” were regarded as more enjoyable (mean values between 4.8 and 5.3) than opera, religious music, and folk/country music (mean values between 3.1 and 3.6). Thus the mean results all appear in the lower half of the scales.

Only one participant was playing an instrument at the time the experiments took place. Three participants stated that they played an instrument in childhood quite often, whereas five of the subjects did not. Eight out of ten reported about frequently listening to music after implantation.

### 3.2. Listening Experiments

**Figure 6:**
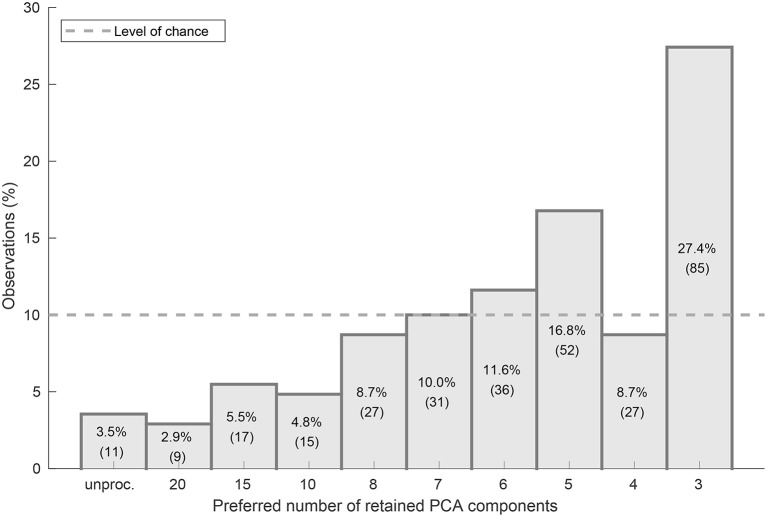
Histogram plot of the numbers of observations per complexity reduction level in percent of overall 310 observations. The level of chance is indicated by the dashed horizontal line.

The listening experiments exhibit a significant preference for music signal excerpts with a high spectral complexity reduction level (median of 5 retained PCA components) as shown in Figure 6. More than half of the ratings of all subjects and music pieces belong to the spectral complexity reduction levels 8, 9, and 10 which correspond to 5, 4, and 3 retained components, respectively.

To investigate the deviation from the level of chance, a two-sided Wilcoxon rank-sum test on the overall listening experiment results was performed under the null hypothesis that the overall preference ratings were uniformly distributed with a probability of 10% for all ten available complexity reduction levels. The null hypothesis was rejected with a *p*-value of *p* ≤ 0.001, indicating that the experimental result significantly deviated from the level of chance (see Figure 6).

**Figure 7:**
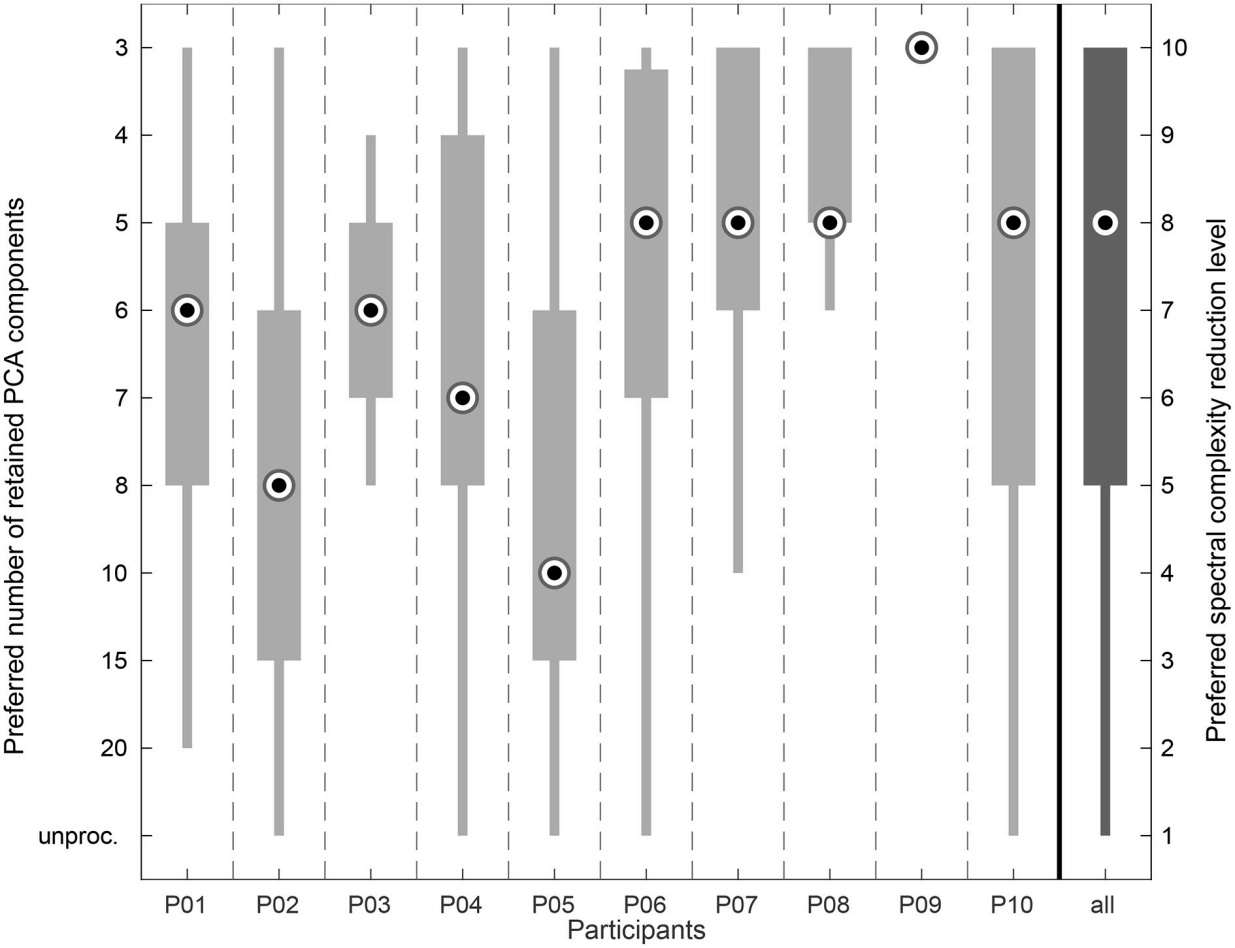
Box plot showing the listening experiment results accumulated over all sessions per participant. The very right column shows the overall distribution of the preference ratings.

A high interindividual variability in the preference ratings could be observed (see Figure 7). Subject P09 preferred the highest spectral complexity reduction level for every piece in every session. Regarding the remaining participants, with interquartile ranges (IQR) covering 2 and 3 spectral complexity reduction levels, respectively, the ratings of subjects P03 and P08 and of subjects P01 and P07 were the least scattered. Subject P10 showed the highest variation in her preference ratings with an IQR covering 5 spectral complexity reduction levels. Regarding the complete data, the IQR covered the range between the complexity reduction levels 5–10 that correspond to a range between 8 and 3 retained components.

**Figure 8:**
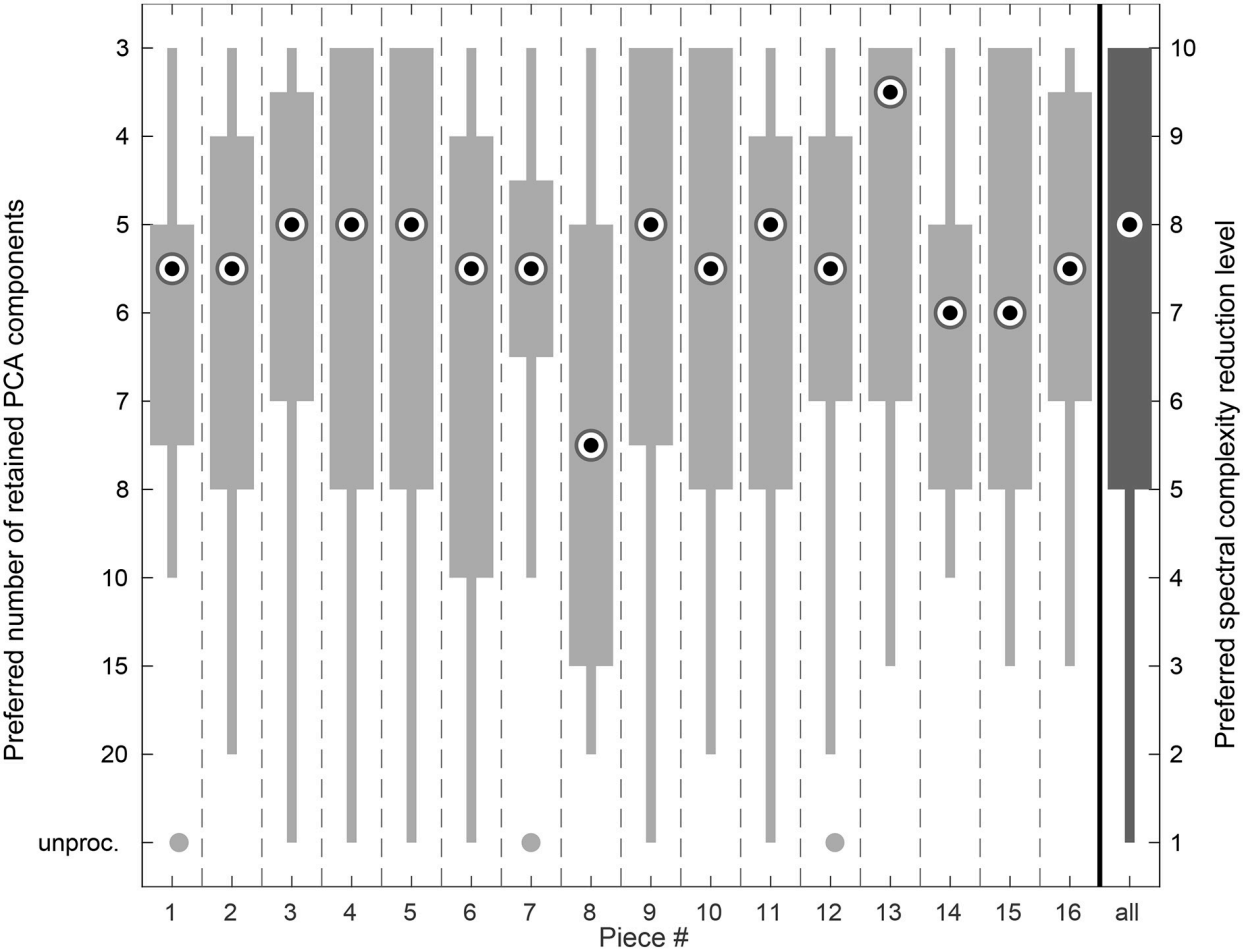
Box plot showing the listening experiment results accumulated over all sessions per piece. The very right column shows the overall distribution of the preference ratings.

Figure 8 shows the preference ratings for each piece. The median values for each piece ranged between 7.5 and 3.5 retained PCA components in the spectrally reduced signals. The interquartile ranges varied between 2 (piece #7) and 5 out of 10 complexity reduction levels (pieces #4, #5, #6, #8, #10, #15). For piece #8 most of the participants preferred a noticeable higher number of retained PCA components (median 7.5 compared to median 5 components overall), whereas for piece #13 a considerably lower number of retained PCA components (median of 3.5) was preferred.

**Figure 9:**
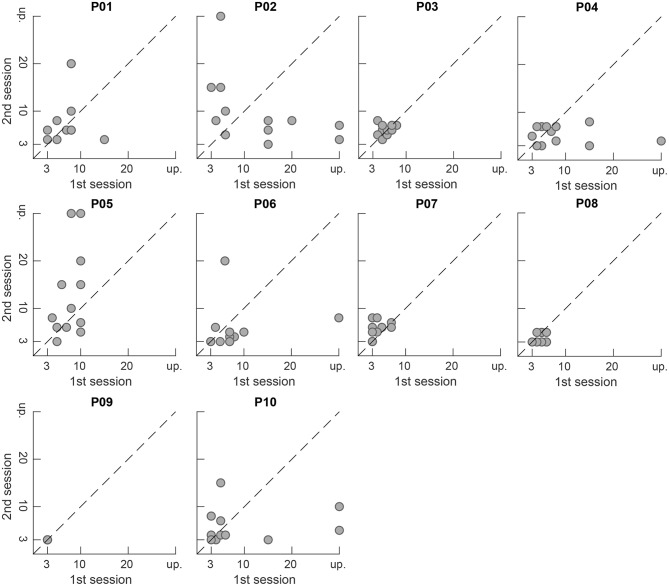
Preferred number of retained components in the second session plotted vs. the preferred number in the first session per participant. Points close to the dashed diagonal line indicate equal ratings in the first and second presentation of a piece. Unprocessed signals are denoted by “up”.

**Figure 10:**
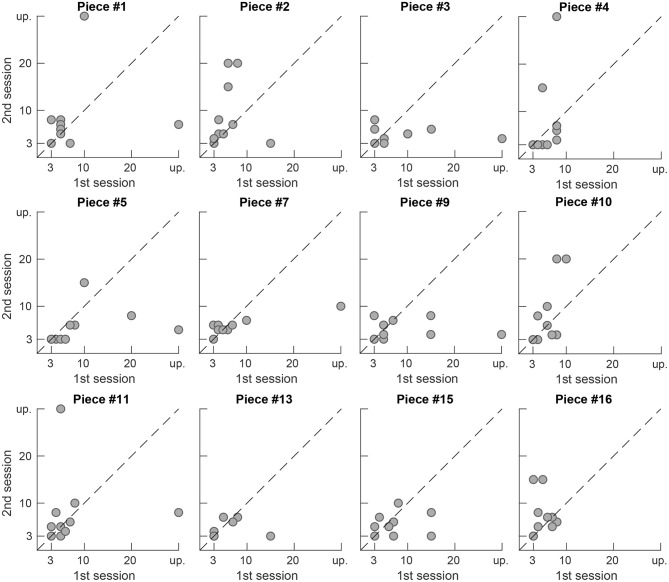
Preferred number of retained components in the second session plotted vs. the preferred number in the first session per piece. Points close to the dashed diagonal line indicate equal ratings in the first and second presentation of a piece. Unprocessed signals are denoted by “up”.

Figures 9, 10 show the preferred number of retained PCA components in the second presentation of a piece plotted vs. the number in the first presentation for each participant and for each piece, respectively. Markers close to the dashed diagonal line indicate that the participants preferred a similar level of complexity reduction in the first and the second presentation of a piece. Markers above the line indicate a preference for a lower level of complexity reduction and thus a less processed version in the second presentation and vice versa. While subjects P03, P07, and P08 widely preferred equal complexity reduction levels in both sessions, for subjects P02 and P10 a higher variation between the preferred values in both session can be observed. Subject P09 preferred maximum complexity reduction (level 10, 3 retained components) in every trial and thus exhibits no tendency over time. Note that this representation is not available for the pieces #6, #8, #12, and #14 as they have only been presented once during the whole study (compare the scheme depicted in Figure 2 for the distribution of the pieces to particular sessions). We applied a Wilcoxon signed-rank test to the results from the first and second presentations which is not significant (α = 0.05) and therefore does not reject the null hypothesis that the data from these two samples have equal medians neither for the overall data (*p* = 0.62) nor for any participant (*p* ≥ 0.06) nor for any piece (*p* ≥ 0.09).

**Figure 11:**
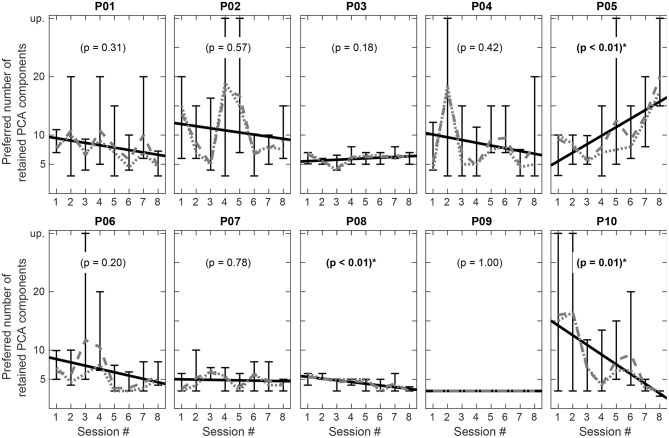
Preferred number of retained components in each session per participant. Vertical bars represent the overall range of ratings in the particular session, dashed and dotted lines show the mean and median for each session. The black solid line shows a linear regression over all sessions. A positive gradient of the regression line indicates a tendency toward lower, a negative gradient toward higher complexity reduction over time. Statistically significant regressions are indicated by (*), unprocessed signals are denoted by “up”.

Figure 11 shows the preferred number of retained components per participant plotted against the listening experiment sessions and thus their development over time during the course of the study. The vertical bars comprise the total range of values observed during the particular sessions. Furthermore, the dotted and dashed lines show the median and the mean values for each session. A linear regression with the session index as predictor was performed and the resulting regression lines for each participant are also depicted in Figure 11. As the sessions are consecutive in time, the gradients of the regression lines illustrate whether the subjects exhibit a tendency either toward higher or toward lower complexity reduction levels during the course of the study. A rising line toward higher numbers of retained components thus indicates a tendency toward an increasing preference for more spectral complexity over time, whereas a falling line toward smaller numbers of retained components indicates an increasing preference for stronger complexity reduction.

### 3.3. Instrumental Assessment of Spectral Spread Improvement

**Figure 12:**
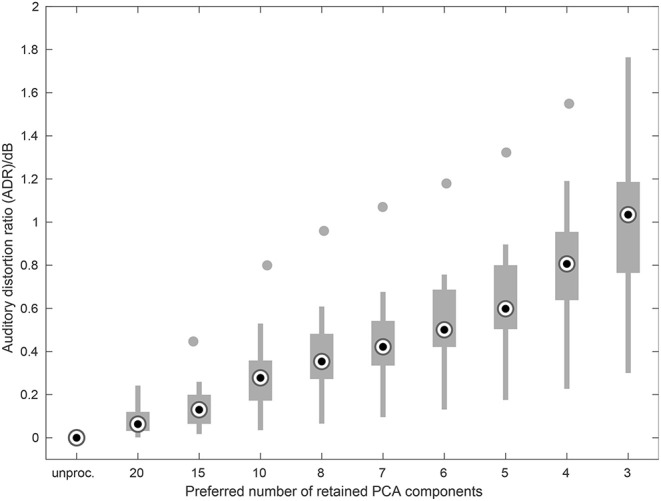
Auditory Distortion Ratio (ADR) plotted vs. the number of retained PCA components averaged over all pieces used in the listening experiments. Higher values indicate a decrease in spectral spread. Filled circles denote outliers.

Figure 12 shows the Auditory Distortion Ratio (ADR) measure averaged over the database of chamber music excerpts for the numbers of retained PCA components used in this study. Positive ADR values indicate a reduction of auditory distortion. The steady increase in ADR with decreasing number of retained components up to a median value of 1 dB and a maximum value of 1.7 dB indicates a reduction in auditory distortion for the spectrally reduced music signals.

The distribution of the preference ratings per complexity level (see Figure 6) is significantly correlated with the ADR values for the respective complexity reduction levels. For the overall data a correlation coefficient of ρ = 0.88 (*p* < 0.001) is found. Hence, the participants in the listening experiments significantly preferred signals that have been processed in such a way that the effects of reduced frequency selectivity, as predicted by the Auditory Distortion Ratio measure, are minimized.

### 3.4. Relations Between Listening Experiments and Questionnaire Results

A correlation analysis between the median results of the listening experiments for each participant and the main questionnaire scores yields correlation coefficients of ρ = −0.19 for the HISQUI (*p* = 0.60), ρ = 0.51 for the Gold-MSI General Musical Sophistication (*p* = 0.13) and ρ = 0.47 for the Mini-PROMS Total scores (*p* = 0.17), respectively. Additionally, for the correlation of the questionnaire scores among each other we found a maximum correlation coefficient of ρ = 0.62 (*p* = 0.057) between the Gold-MSI General Musical Sophistication and the Mini-PROMS Total scores. However, with *p* > 0.057 none of these correlations are significant with regard to α = 0.05.

## 4. Discussion

In this section we will first discuss the outcomes of the listening experiments with regard to the preferred amount of spectral complexity reduction. Subsequently we will investigate the impact of potential influencing factors and possible predictors: the selection of stimuli, the etiology, individual CI parameters, or the self-reported sound quality and musical ability assessments provided by the questionnaires.

### 4.1. Listening Experiments

All participants of the presented study preferred a spectral complexity reduction of 8 or even less retained PCA components with a median of 5 components (see Figure 6). With reduced spectral complexity the information from a musical piece is concentrated on a smaller number of frequency bands. Thus, signals with a reduced spectral complexity induce less broad excitation patterns and are therefore supposed to be more intelligible and accessible for CI listeners as the effect of spectral spread is reduced. The processed signals from the chamber music database used in the listening experiments exhibit a lower spectral complexity as both the harmonic components of the leading voice and the accompanying voices are attenuated. Hence the preference for a high spectral complexity reduction found in the participating CI users is in line with the findings by Nemer et al. (2017) where CI users reported an increase in enjoyment of monophonic pieces with a reduced overtone series, and the findings by Kohlberg et al. (2015) where CI users rated recordings of pieces with a reduced number of competing instruments as more pleasant than the original. But in contrast to the method used by Nemer et al., the PCA-based spectral complexity reduction scheme does not reduce the overtone series in an ordered fashion from higher to lower order toward the fundamental frequency. It identifies and preserves those spectral components with the highest signal power, thus, spectral regions with lower power are attenuated.

Nagathil et al. (2016) and Nagathil et al. (2017) reported preference scores of up to 73.7% (CI listeners) and 75.6% (NH listeners with spectral smearing) for the spectrally reduced stimuli compared to the unprocessed signals in listening experiments using the same spectral complexity reduction scheme. Those results, however, cannot be directly compared to the outcome of the study at hand as these studies used a different experimental design and examined a more heterogeneous group of participants. Nevertheless, those earlier findings were generally confirmed. Moreover, in most of the cases the participants in this study preferred an even stronger reduction of spectral complexity.

In contrast to the earlier studies where the stimuli were presented under free field conditions, bimodal listening (CI on one side and HA on the other) was excluded in this study. This might have led to a preference for higher spectral complexity reduction levels as listeners who rely solely on electrical hearing are supposed to experience a higher impact of spectral spread than those with additional acoustic hearing. This is in line with studies by Gfeller et al. (2006) showing that bimodal listeners rate music more pleasant than listeners relying only on electric stimulation.

The preference ratings depicted in the histogram plot in Figure 6 tend to rise with decreasing number of retained components. However, complexity reduction level 9 with 4 retained PCA components has been preferred surprisingly more rarely than the adjacent levels with 5 and 3 components. This can be partly explained by subject P09 who exhibited a monotonic preference for complexity reduction level 10 with 3 retained PCA components and thus disproportionately contributed to the overall number of preference ratings for this complexity level. However, even if we would leave out all judgments by this subject the remaining 54 observations for complexity reduction level 10 would still be approximately equal to those for level 8 (5 components) and thus show a noticeable drop at level 9. The perceptual differences between particular spectral complexity reduction levels increase with decreasing number of retained PCA components, at least for normal-hearing listeners. The participants also confirmed this impression informally during the experiments. Therefore, we assume that the participants regularly were not able to notice a sufficient difference between the signals with levels 8 and 9 or 9 and 10. We rather suppose that the participants in doubt chose the maximum complexity reduction and therefore introduced a certain amount of bias at this upper limit of the complexity reduction scale.

Listening experiments with CI listeners concerning music perception usually exhibit higher amounts of variation. This affects both tests with regard to music perception in general or comparison tests with modified music signals (compare e.g., Gfeller et al., 2000, Gfeller et al., 2002, Gfeller et al., 2003 and Wright and Uchanski, 2012 or Roy et al., 2012, Kohlberg et al., 2015 and Pons et al., 2016). Furthermore, the pieces in the particular database differ in their musical characterics and feature different instruments, tempi and rhythms. Therefore, a certain amount of interindividual and intraindividual variation in the preference ratings as well as between the pieces is generally plausible and to be expected.

As stated before, subject P09 preferred the maximum spectral complexity reduction in all sessions and for all pieces. This might be caused by the fact that this participant is the only one suffering from a hearing loss since adolescence (see Table 1) and had significantly lower speech intelligibility scores than the other participants of only 40% (vs. mean of 61%) at 65 dB and of 50% (vs. mean 80%) at 80 dB, respectively. Her score in the HISQUI questionnaire, however, corresponds to a “good sound quality” rating.

The piecewise representation of the preferred spectral complexity reduction levels in Figure 8 shows considerable deviations for two pieces. For piece #8 the participants preferred a higher number of retained PCA components (median 7.5 compared to 5 overall), for piece #13 a smaller number of components (median 3.5) and thus a higher level of spectral complexity reduction was preferred. A possible explanation for piece #8 might be the fact that the accompaniment in this excerpt is played in the same rhythm as the melody and hence both coincide in the most prominent PCA components. For normal-hearing listeners the difference between the respective spectrally reduced versions of this particular piece is therefore rather small for most of the spectral complexity reduction levels. For piece #13, however, a comparable explanation cannot be found.

In Figures 9, 10 the accumulation of data points close to the main diagonal shows that the majority of participants come to a similar judgment when listening to the same musical piece twice. For subjects P03, P07 and P08 the data points are clearly clustered close to the main diagonal, as well as for P01 and P06 which, however, each exhibit two outliers (out of 12 repeatedly presented pieces in total). For some participants (P02, P05) and some pieces (#2, #5) slight variations between the first and the second presentation of the signals in different sessions can be observed in the figures. Nevertheless, as mentioned earlier in section 3.2, a Wilcoxon signed rank test (α = 0.05) did not reject the null hypothesis that the samples from the first and the second presentation were drawn from populations having the same distribution.

Considering the intraindividual variation in preference ratings over time in Figure 11, four out of ten participants (P03, P07, P08, P09) did not show large changes in between the eight sessions analyzed with regard to the preferred spectral complexity reduction level, whereas in six subjects larger variations could be detected. As mentioned above, our music perception experiments showed no evidence for a general habituation or adaption effect with regard to the spectral complexity reduction level. Therefore, our research hypothesis that repeated engagement with music signals of reduced spectral complexity facilitates the access to signals with higher complexity needs to be rejected at the moment. Nevertheless, statistically significant tendencies for a gradual change of the preferred spectral complexity reduction level could be found in the regression analysis for the subjects P05 (*p* ≤ 0.01), P08 (*p* ≤ 0.01), and P10 (*p* = 0.01). While in general most of the subjects showed a slight tendency toward a stronger complexity reduction in the follow-up, only two participants (P03, P05) showed a tendency toward a weaker spectral complexity reduction at the end of the study period. Hence, these tendencies could still be an indication for a habituation effect with regard to spectral complexity reduction, at least in these subjects. In earlier studies where different music preprocessing schemes for cochlear implant users were evaluated, intraindividual results vary rather strong (Pons et al., 2016; Gajęcki and Nogueira, 2018). Also, in these studies the long-term development of preference ratings for different parameter setting was not investigated. Furthermore, music enjoyment and thus music perception tests, especially with CI listeners, typically exhibit a higher variability in the subjects' assessments, as there is a wide range of additional factors which might have an influence on musical enjoyment: The enjoyment of music depends—beside the structural features of the music—also on the emotional and mental state and the expectations of the listeners. Even the time of the day can have an additional impact. This might also partly explain the high variability in music perception studies with CI listeners.

As Fuller et al. (2018) stated, there is only little knowledge about the effects of long-term music training on auditory, music, and speech perception. This also holds in regard to the required observation period for training, adaption or habituation effects. Several studies point out that even a short music training might enhance perceptual accuracy on some aspects of music, such as pitch discrimination (Vandali et al., 2015), melodic contour recognition or timbre (Driscoll, 2012; Galvin et al., 2012) and melody recognition appraisal or general enjoyment. However, the degree of benefit differs considerably among the CI users (Driscoll et al., 2009; Galvin et al., 2012; Looi et al., 2012; Gfeller et al., 2015). The underlying reason might be the kind of training used but also physiological factors, such as the survival of auditory neurons. According to prevalent theories about the optimal complexity level between stimulation and overtaxing. Gfeller et al. (2003) stated that CI listeners prefer a lower level than normal-hearing subjects. This finding could be confirmed by Nagathil et al. (2018). Hence, possible adaption or habituation processes as indicated in some of the participants in this study would need to be further investigated over a longer observation period than 4 weeks. While Fuller et al. (2018) chose a 6-weeks observation period, Vandali et al. (2015) observed effects of musical training after cochlear implantation for 4 months and Petersen et al. (2012) even for 6 months.

### 4.2. Influencing Factors With Potential Impact on the Outcomes

A Mann–Whitney *U* test showed no significant factor effects for any of the musical factors “mean interval size” (MIS, *p* = 0.90), “mean fundamental frequency” (MFF, *p* = 0.97) and “mean tone duration” (MTD, *p* = 0.81) as inferred from the Plackett-Burman experimental design. This outcome is in agreement with earlier evaluations of spectral complexity reduction methods by Nagathil et al. (2016) and Nagathil et al. (2017) where statistically significant factor effects could only be found for the additional methods (PLS, ASNA) investigated alongside the PCA-based method. These musical factors therefore still show no influence although the range of the spectral complexity reduction parameter was considerably extended in the current study.

#### 4.2.1. Questionnaire Results

The comparatively poor results from the Mini-PROMS test (see section 3.1.3) indicate that pitch-related tasks like melody recognition are quite difficult for the majority of the CI recipients included in this study. A correlation analysis, though, indicated no significant relation between the median preference ratings on the one hand and the subject-related data and questionnaire results like the perceived sound quality (HISQUI, *p* = 0.61), the music sophistication (Gold-MSI, *p* = 0.13), the musical abilities (Mini-PROMS, *p* = 0.17), and the listening habits (Munich Music Questionnaire, *p* ≥ 0.10) on the other hand. Therefore, the hypothesis that participants with high scores in the questionnaires might prefer a higher spectral complexity and thus a higher number of retained PCA components cannot be confirmed. A comparison of the results from the questionnaires and the listening experiments for participant P08 illustrates this: This subject obtained the maximum scores in all questionnaires (see Figure 4). It could be expected that such results would predict a preference for weaker spectral complexity reduction. However, in contrast this participant mostly preferred a rather small number of PCA components (median of 5 components) and only a small variation with an IQR covering the range between 5 and 3 components (see Figure 7). In the earlier study by Nagathil et al. (2017) a similar effect could be observed in one participant who reported to perform a regular music training and exhibited a considerably stronger preference for higher spectral complexity reduction (8 instead of 13 retained PCA components). Therefore, we advance the hypotheses that CI listeners with a higher degree of musical training or listening experience might prefer a higher degree of spectral complexity reduction because the spectrally reduced auditory input enables them to benefit even more from their abilities and training achievements than the unprocessed sounds. This hypothesis would be a promising subject for future research.

#### 4.2.2. CI Parameters

Although this was none of the primary research questions in this study, we also analyzed a possible relation between the preferred spectral complexity reduction levels and some individual CI parameters like the filterbank configuration and the estimated tonotopic position of the three most apical electrodes (see Table 2). A significant correlation between the individual filterbank configuration and the preferred spectral complexity reduction level could not be observed (*p* ≥ 0.43).

According to recent studies e.g., by Hochmair et al. (2015), a combination of fine structure preserving stimulation strategies and a deep insertion of the electrode array toward the apical region results in improved music enjoyment. Hence we additionally took the insertion depth of the implants into the cochlea into account. For five of the participants in this study (P01, P03, P06, P09, P10) multiplanar reconstructed CT scan images were available. For these subjects the cochlear duct length was estimated based on the measurement of two diameters of the basal turn of the cochlea (Koch et al., 2017, Alexiades et al., 2015) using the OTOPLAN^3^ otological surgery planning software (CASCINATION AG, Bern, Switzerland). Based on this length in turn the tonotopically corresponding frequencies at the positions of the first three electrodes were approximated (see Table 2). For the remaining five subjects (P02, P04, P05, P07, P08) the quality of the CT images was not sufficient or the data were not available. In subjects P06 and P10 the cochlea is not completely covered by the electrode, so that frequencies below *f*_min_≈400 Hz cannot be stimulated adequately. In subjects P01 and P09 and even more in subject P03 the implanted electrode is covering almost the whole length of the cochlea and thus also the apical part which is responsible for the low frequencies. For subject P03 where the implanted electrode covers the cochlear duct almost completely we observe a comparatively small variation between the preferred spectral complexity reduction levels, whereas subjects P06 and P10 exhibit a wider variation comprising all available complexity reduction levels. However, no significant correlations between the estimated tonotopic frequencies at the electrode positions and the spectral complexity preference ratings were observed (*p* ≥ 0.74).

The only two subjects using the HiRes stimulation strategy (P04 and P05) both preferred a larger number of retained PCA components and thus less spectral complexity reduction (median of 7 and 10 retained components compared to a median of 5 components in the overall data). Furthermore, subject P07 who also has a filterbank configuration tuned to higher center frequencies (see Table 2) preferred a smaller number of retained PCA components (median of 5) that coincides with the overall data. However, the number of subjects examined in this study is too small to draw reliable conclusions on the relationship between the individual filterbank configurations, the cochlear coverage by the implant or the use of a fine structure coding strategy on the one hand and the preferred spectral complexity reduction levels on the other hand. Therefore, these relations should also be investigated in future studies.

### 4.3. Conclusions

Many of the limitations for music perception in CI users are due to the coarse electric-neural interface of current CIs. As major changes in electrode design are not to be expected in the short run, we consider signal preprocessing techniques, besides additional rehabilitation and training efforts, to be the major means in facilitating music appraisal in CI users. The found preference for signals with a reduced number of retained PCA components is in line with the evaluation of other preprocessing methods where the spectral complexity was reduced by reducing the number of overtones manually (Nemer et al., 2017) or by remixing music signals to enhance the leading voices, vocals and rhythmic components (Kohlberg et al., 2015, Pons et al., 2016, Buyens et al., 2017, Gajęcki and Nogueira, 2018). The evaluated method in the study at hand directly tackles the harmonic and pitch-related features of musical signals to reduce the impact of spectral spread in CI listeners. In future work the spectral complexity reduction will be complemented by techniques modifying the rhythmic and percussive portions of music signals in order to obtain a comprehensive preprocessing strategy.

## Data Availability Statement

The datasets generated for this study are available on request from the corresponding author.

## Ethics Statement

This study was carried out in accordance with the recommendations of the ICH-GCP guidelines and the respective national legal provisions in Germany. All subjects gave written informed consent in accordance with the Declaration of Helsinki. The protocol was approved by the Ethics Committee of the medical faculty of the Ruhr-Universität Bochum (Ethik-Kommission der Medizinischen Fakultät der Ruhr-Universität Bochum, registration number 16-5998).

## Author Contributions

CV, JG, and AN designed the study. CV and JT selected the subjects and provided medical attendance. JG and AN developed the signal processing scheme and the interactive user interface and conducted the listening experiments. RM contributed to the signal processing scheme. JG, CV, and AN analyzed and evaluated the data. JG wrote the manuscript with contributions and critical feedback from all authors. RM, CV, and JT supervised the project.

### Conflict of Interest

The authors declare that the research was conducted in the absence of any commercial or financial relationships that could be construed as a potential conflict of interest.

## Footnotes

^1^https://www.medel.com/support/sonnet/connectivity/sonnet_direct_audio_input

^2^https://advancedbionics.com/us/en/home/products/accessories/wireless-media-connectivity.html

^3^https://www.otoplan.ch/

## References

[B1] Alexiades G. Dhanasingh A. Jolly C. (2015). Method to estimate the complete and two-turn cochlear duct length. Otol. Neurotol. 36, 904–907. 10.1097/MAO.0000000000000620 25299827

[B2] Amann E. Anderson I. (2014). Development and validation of a questionnaire for hearing implant users to self-assess their auditory abilities in everyday communication situations: the Hearing Implant Sound Quality Index (HISQUI19). Acta Otolaryngol. 134, 915–923. 10.3109/00016489.2014.909604 24975453

[B3] Brockmeier S. J. Grasmeder M. Passow S. Mawmann D. Vischer M. Jappel A.. (2007). Comparison of musical activities of cochlear implant users with different speech-coding strategies. Ear Hear. 28:49S. 10.1097/AUD.0b013e3180315468 17496646

[B4] Brown J. C. (1991). Calculation of a constant Q spectral transform. J. Acoust. Soc. Am. 89, 425–434.

[B5] Bruns L. Mürbe D. Hahne A. (2016). Understanding music with cochlear implants. Sci. Rep. 6:32026. 10.1038/srep32026 27558546 PMC4997320

[B6] Buyens W. van Dijk B. Moonen M. Wouters J. (2014). Music mixing preferences of cochlear implant recipients: a pilot study. Int. J. Audiol. 53, 294–301. 10.3109/14992027.2013.873955 24471410

[B7] Buyens W. van Dijk B. Moonen M. Wouters J. (2017). Evaluation of a stereo music preprocessing scheme for cochlear implant users. J. Am. Acad. Audiol. 29, 35–43. 10.3766/jaaa.16103 29309022

[B8] Buyens W. van Dijk B. Wouters J. Moonen M. (2015). A stereo music preprocessing scheme for cochlear implant users. IEEE Trans. Biomed. Eng. 62, 2434–2442. 10.1109/TBME.2015.2428999 25966469

[B9] Caldwell M. T. Jiradejvong P. Limb C. J. (2016). Impaired perception of sensory consonance and dissonance in cochlear implant users. Otol. Neurotol. 37:229. 10.1097/MAO.0000000000000960 26825669

[B10] Cappotto D. Xuan W. Meng Q. Zhang C. Schnupp J. (2018). Dominant melody enhancement in cochlear implants, in 2018 Asia-Pacific Signal and Information Processing Association Annual Summit and Conference (APSIPAASC) (Honolulu, HI), 398–402.

[B11] Donnelly P. J. Guo B. Z. Limb C. J. (2009). Perceptual fusion of polyphonic pitch in cochlear implant users. J. Acoust. Soc. Am. 126, EL128–EL133. 10.1121/1.3239464 19894787

[B12] Driscoll V. D. (2012). The effects of training on recognition of musical instruments by adults with cochlear implants. Semin. Hear. 33, 410–418. 10.1055/s-0032-1329230 23503992 PMC3595548

[B13] Driscoll V. D. Oleson J. Jiang D. Gfeller K. (2009). Effects of training on recognition of musical instruments presented through cochlear implant simulations. J. Am. Acad. Audiol. 20, 71–82. 10.3766/jaaa.20.1.7 19927684 PMC2784659

[B14] Fuller C. D. Galvin J. J. Maat B. Başkent D. Free R. H. (2018). Comparison of two music training approaches on music and speech perception in cochlear implant users. Trends Hear. 22:2331216518765379. 10.1177/2331216518765379 29621947 PMC5894911

[B15] Gajęcki T. Nogueira W. (2018). Deep learning models to remix music for cochlear implant users. J. Acoust. Soc. Am. 143, 3602–3615. 10.1121/1.5042056 29960485

[B16] Galvin J. Eskridge E. Oba S. Fu Q.-J. (2012). Melodic contour identification training in cochlear implant users with and without a competing instrument. Semin. Hear. 33, 399–409. 10.1055/s-0032-1329227

[B17] Galvin J. J. Fu Q.-J. Shannon R. V. (2009). Melodic contour identification and music perception by cochlear implant users. Ann. N. Y. Acad. Sci. 1169, 518–533. 10.1111/j.1749-6632.2009.04551.x 19673835 PMC3627487

[B18] Gauer J. Nagathil A. Martin R. (2018). Binaural spectral complexity reduction of music signals for cochlear implant listeners, in Proceedings of IEEE International Conference on Acoustics, Speech and Signal Processing (ICASSP) (Calgary, AB: IEEE), 251–255.

[B19] Gfeller K. Christ A. Knutson J. Witt S. Mehr M. (2003). The effects of familiarity and complexity on appraisal of complex songs by cochlear implant recipients and normal hearing adults. J. Music Ther. 40, 78–112. 10.1093/jmt/40.2.78 14505444

[B20] Gfeller K. Christ A. Knutson J. F. Witt S. Murray K. T. Tyler R. S. (2000). Musical backgrounds, listening habits, and aesthetic enjoyment of adult cochlear implant recipients. J. Am. Acad. Audiol. 11, 390–406. 10976500

[B21] Gfeller K. Guthe E. Driscoll V. Brown C. J. (2015). A preliminary report of music-based training for adult cochlear implant users: rationales and development. Cochlear Implants Int. 16, S22–S31. 10.1179/1467010015Z.000000000269 26561884 PMC4646703

[B22] Gfeller K. Oleson J. Knutson J. F. Breheny P. Driscoll V. Olszewski C. (2008). Multivariate predictors of music perception and appraisal by adult cochlear implant users. J. Am. Acad. Audiol. 19, 120–134. 10.3766/jaaa.19.2.3 18669126 PMC2677551

[B23] Gfeller K. Witt S. Mehr M. A. Woodworth G. Knutson J. (2002). Effects of frequency, instrumental family, and cochlear implant type on timbre recognition and appraisal. Ann. Otol. Rhinol. Laryngol. 111, 349–356. 10.1007/BF02105664 11991588

[B24] Gfeller K. E. Olszewski C. Turner C. Gantz B. Oleson J. (2006). Music perception with cochlear implants and residual hearing. Audiol. Neurootol. 11, 12–15. 10.1159/000095608 17063005

[B25] Hahlbrock K.-H. (1953). Über Sprachaudiometrie und neue Wörterteste. Archiv. Ohren Nasenu. Kehlkopfheil. 162, 394–431. 13092895

[B26] Hall D. A. Johnsrude I. S. Haggard M. P. Palmer A. R. Akeroyd M. A. Summerfield A. Q. (2002). Spectral and temporal processing in human auditory cortex. Cereb. Cortex 12, 140–149. 10.1093/cercor/12.2.140 11739262

[B27] Hochmair I. Hochmair E. Nopp P. Waller M. Jolly C. (2015). Deep electrode insertion and sound coding in cochlear implants. Hear. Res. 322, 14–23. 10.1016/j.heares.2014.10.006 25456089

[B28] Innes-Brown H. Au A. Stevens C. Schubert E. Marozeau J. (2012). New music for the bionic ear: an assessment of the enjoyment of six new works composed for cochlear implant recipients, in Proceedings of 12th International Conference on Music Perception and Cognition (ICMPC) 8th Triennial Conference of the European Society for the Cognitive Sciences of Music (ESCOM) (Thessaloniki), 482–491.

[B29] Jiam N. T. Caldwell M. T. Limb C. J. (2017). What does music sound like for a cochlear implant user? Otol. Neurotol. 38, e240–e247. 10.1097/MAO.0000000000001448 28806333

[B30] Koch R. W. Ladak H. M. Elfarnawany M. Agrawal S. K. (2017). Measuring cochlear duct length–a historical analysis of methods and results. J. Otolaryngol. Head Neck Surg. 46:19. 10.1186/s40463-017-0194-2 28270200 PMC5341452

[B31] Kohlberg G. D. Mancuso D. M. Chari D. A. Lalwani A. K. (2015). Music engineering as a novel strategy for enhancing music enjoyment in the cochlear implant recipient. Behav. Neurol. 2015, 1–7. 10.1155/2015/829680 26543322 PMC4620405

[B32] Lassaletta L. Castro A. Bastarrica M. Pérez-Mora R. Madero R. De Sarriá J.. (2007). Does music perception have an impact on quality of life following cochlear implantation? Acta Otolaryngol. 127, 682–686. 10.1080/00016480601002112 17573562

[B33] Law L. N. C. Zentner M. (2012). Assessing musical abilities objectively: construction and validation of the profile of music perception skills. PLoS ONE 7:e52508. 10.1371/journal.pone.0052508 23285071 PMC3532219

[B34] Lenarz T. (2018). Cochlear implant–state of the art. GMS Curr. Top. Otorhinolaryngol. Head Neck Surg. 16:Doc04. 10.3205/cto000143 29503669 PMC5818683

[B35] Limb C. J. Roy A. T. (2014). Technological, biological, and acoustical constraints to music perception in cochlear implant users. Hear. Res. 308, 13–26. 10.1016/j.heares.2013.04.009 23665130

[B36] Looi V. Gfeller K. Driscoll V. (2012). Music appreciation and training for cochlear implant recipients: a review. Semin. Hear. 33, 307–334. 10.1055/s-0032-1329222 23459244 PMC3583543

[B37] Looi V. McDermott H. McKay C. Hickson L. (2004). Pitch discrimination and melody recognition by cochlear implant users. Int. Congr. Ser. 1273, 197–200. 10.1016/j.ics.2004.08.038

[B38] Looi V. McDermott H. McKay C. Hickson L. (2007). Comparisons of quality ratings for music by cochlear implant and hearing aid users. Ear Hear. 28:59S. 10.1097/AUD.0b013e31803150cb 17496649

[B39] McDermott H. J. (2004). Music perception with cochlear implants: a review. Trends Amplif. 8, 49–82. 10.1177/108471380400800203 15497033 PMC4111359

[B40] Moore B. C. J. Glasberg B. R. Simpson A. (1992). Evaluation of a method of simulating reduced frequency selectivity. J. Acoust. Soc. Am. 91, 3402–3423. 1619117 10.1121/1.402830

[B41] Müllensiefen D. Gingras B. Musil J. Stewart L. (2014). The musicality of non-musicians: an index for assessing musical sophistication in the general population. PLoS ONE 9:e89642. 10.1371/journal.pone.0089642 24586929 PMC3935919

[B42] Müller J. Brill S. Hagen R. Moeltner A. Brockmeier S.-J. Stark T.. (2012). Clinical trial results with the MED-EL fine structure processing coding strategy in experienced cochlear implant users. ORL J. Otorhinolaryngol. Relat. Spec. 74, 185–198. 10.1159/000337089 22814383

[B43] Müller-Deile J. (2009). [Speech intelligibility tests in cochlear implant patients]. HNO 57, 580–592. 10.1007/s00106-009-1930-3 19517086

[B44] Nagathil A. Martin R. (2012). Optimal signal reconstruction from a constant-Q spectrum, in IEEE International Conference on Acoustics, Speech and Signal Processing (ICASSP) (Kyoto), 349–352.

[B45] Nagathil A. Schlattmann J.-W. Neumann K. Martin R. (2018). Music complexity prediction for cochlear implant listeners based on a feature-based linear regression model. J. Acoust. Soc. Am. 144, 1–10. 10.1121/1.5044514 30075690

[B46] Nagathil A. Weihs C. Martin R. (2016). Spectral complexity reduction of music signals for mitigating effects of cochlear hearing loss. IEEE ACM Trans. Audio Speech Lang. Process. 24, 445–458. 10.1109/TASLP.2015.2511623

[B47] Nagathil A. Weihs C. Neumann K. Martin R. (2017). Spectral complexity reduction of music signals based on frequency-domain reduced-rank approximations: an evaluation with cochlear implant listeners. J. Acoust. Soc. Am. 142, 1219–1228. 10.1121/1.5000484 28964082

[B48] Nemer J. S. Kohlberg G. D. Mancuso D. M. Griffin B. M. Certo M. V. Chen S. Y.. (2017). Reduction of the harmonic series influences musical enjoyment with cochlear implants. Otol. Neurotol. 38, 31–37. 10.1097/MAO.0000000000001250 27755358 PMC5154854

[B49] Nogueira W. Herrera P. (2015). Creation and analysis of new music compositions for cochlear implant users, in 18. Jahrestagung Der Deutschen Gesellschaft Für Audiologie (DGA), Bochum.

[B50] Nogueira W. Nagathil A. Martin R. (2019). Making music more accessible for cochlear implant listeners: recent developments. IEEE Signal Process. Mag. 36, 115–127. 10.1109/MSP.2018.2874059

[B51] Omran S. A. Lai W. Büchler M. Dillier N. (2011). Semitone frequency mapping to improve music representation for nucleus cochlear implants. EURASIP J. Audio Speech Music Process. 2011:2 10.1186/1687-4722-2011-2

[B52] Petersen B. Mortensen M. V. Hansen M. Vuust P. (2012). Singing in the key of life: a study on effects of musical ear training after cochlear implantation. Psychomusicology 22, 134–151. 10.1037/a0031140

[B53] Plackett R. L. Burman J. P. (1946). The design of optimum multifactorial experiments. Biometrika 33, 305–325.

[B54] Pons J. Janer J. Rode T. Nogueira W. (2016). Remixing music using source separation algorithms to improve the musical experience of cochlear implant users. J. Acoust. Soc. Am. 140, 4338–4349. 10.1121/1.4971424 28040023

[B55] Roy A. T. Jiradejvong P. Carver C. Limb C. J. (2012). Assessment of sound quality perception in cochlear implant users during music listening. Otol. Neurotol. 33, 319–327. 10.1097/MAO.0b013e31824296a9 22314920

[B56] Schaal N. K. Bauer A.-K. R. Müllensiefen D. (2014). Der Gold-MSI: Replikation und Validierung eines Fragebogeninstrumentes zur Messung Musikalischer Erfahrenheit anhand einer deutschen Stichprobe. Music. Sci. 18, 423–447. 10.1177/1029864914541851

[B57] Schönwiesner M. Rübsamen R. von Cramon D. Y. (2005). Spectral and temporal processing in the human auditory cortex—revisited. Ann. N. Y. Acad. Sci. 1060, 89–92. 10.1196/annals.1360.051 16597754

[B58] Sucher C. M. McDermott H. J. (2007). Pitch ranking of complex tones by normally hearing subjects and cochlear implant users. Hear. Res. 230, 80–87. 10.1016/j.heares.2007.05.002 17604582

[B59] Todd A. E. Mertens G. Van de Heyning P. Landsberger D. M. (2017). Encoding a melody using only temporal information for cochlear-implant and normal-hearing listeners. Trends Hear. 21:2331216517739745. 10.1177/2331216517739745 29161987 PMC5703098

[B60] Vandali A. Sly D. Cowan R. van Hoesel R. (2015). Training of cochlear implant users to improve pitch perception in the presence of competing place cues. Ear Hear. 36, e1–e13. 10.1097/AUD.0000000000000109 25329372

[B61] Vannson N. Innes-Brown H. Marozeau J. (2015). Dichotic listening can improve perceived clarity of music in cochlear implant users. Trends Hear. 19:2331216515598971. 10.1177/2331216515598971 26316123 PMC4593516

[B62] Virtanen T. Gemmeke J. F. Raj B. (2013). Active-set Newton algorithm for overcomplete non-negative representations of audio. IEEE Trans. Audio Speech Lang. Process. 21, 2277–2289. 10.1109/TASL.2013.2263144

[B63] Wilson B. S. Dorman M. F. (2008). Cochlear implants: a remarkable past and a brilliant future. Hear. Res. 242, 3–21. 10.1016/j.heares.2008.06.005 18616994 PMC3707130

[B64] Wright R. Uchanski R. M. (2012). Music perception and appraisal: cochlear implant users and simulated cochlear implant listening. J. Am. Acad. Audiol. 23, 350–365. 10.3766/jaaa.23.5.6 22533978 PMC3400338

[B65] Zentner M. Strauss H. (2017). Assessing musical ability quickly and objectively: development and validation of the short-PROMS and the mini-PROMS. Ann. N. Y. Acad. Sci. 1400, 33–45. 10.1111/nyas.13410 28704888

